# Influence of Nonpolio Enteroviruses and the Bacterial Gut Microbiota on Oral Poliovirus Vaccine Response: A Study from South India

**DOI:** 10.1093/infdis/jiy568

**Published:** 2018-09-24

**Authors:** Ira Praharaj, Edward P K Parker, Sidhartha Giri, David J Allen, Sophia Silas, R Revathi, Saravanakumar Puthupalayam Kaliappan, Jacob John, Jasmine Helan Prasad, Beate Kampmann, Miren Iturriza-Gómara, Nicholas C Grassly, Gagandeep Kang

**Affiliations:** 1 Division of Gastrointestinal Sciences, Christian Medical College, Vellore, Tamil Nadu, India; 5 Department of Community Health, Christian Medical College, Vellore, Tamil Nadu, India; 2 Department of Infectious Disease Epidemiology, Imperial College London, London, United Kingdom; 6 Department of Paediatrics, St Mary’s Campus, Imperial College London, London, United Kingdom; 3 Department of Pathogen Molecular Biology, Faculty of Infectious and Tropical Diseases, London School of Hygiene and Tropical Medicine, London, United Kingdom; 4 Enteric Virus Unit, Virus Reference Department, Microbiology Services, Public Health England, London, United Kingdom; 7 Centre for Global Vaccine Research, Institute of Infection and Global Health, and National Institute for Health Research Health Protection Research Unit in Gastrointestinal Infection, University of Liverpool, United Kingdom

**Keywords:** Nonpolio enteroviruses, bacterial microbiota, 16S rRNA, OPV, next generation sequencing

## Abstract

**Background:**

Oral poliovirus vaccine (OPV) is less immunogenic in low- or middle-income than in high-income countries. We tested whether bacterial and viral components of the intestinal microbiota are associated with this phenomenon.

**Methods:**

We assessed the prevalence of enteropathogens using TaqMan array cards 14 days before and at vaccination in 704 Indian infants (aged 6–11 months) receiving monovalent type 3 OPV (CTRI/2014/05/004588). Nonpolio enterovirus (NPEV) serotypes were identified by means of VP1 sequencing. In 120 infants, the prevaccination bacterial microbiota was characterized using 16S ribosomal RNA sequencing.

**Results:**

We detected 56 NPEV serotypes on the day of vaccination. Concurrent NPEVs were associated with a reduction in OPV seroconversion, consistent across species (odds ratio [95% confidence interval], 0.57 [.36–.90], 0.61 [.43–.86], and 0.69 [.41–1.16] for species A, B, and C, respectively). Recently acquired enterovirus infections, detected at vaccination but not 14 days earlier, had a greater interfering effect on monovalent type 3 OPV seroresponse than did persistent infections, with enterovirus detected at both time points (seroconversion in 44 of 127 infants [35%] vs 63 of 129 [49%]; *P* = .02). The abundance of specific bacterial taxa did not differ significantly according to OPV response, although the microbiota was more diverse in nonresponders at the time of vaccination.

**Conclusion:**

Enteric viruses have a greater impact on OPV response than the bacterial microbiota, with recent enterovirus infections having a greater inhibitory effect than persistent infections.

## Teaser

Nonpolio enteroviruses have a significant impact on oral poliovirus vaccine seroresponse irrespective of infecting serotype, whereas the bacterial gut microbiota does not affect OPV seroresponse significantly.

**(See the Editorial Commentary by Ramani and Harris on pages 1173–5.)**

Oral vaccines have consistently proved to be less immunogenic in low- and middle-income than in high-income countries [1–3]. Several mechanisms may contribute to this phenomenon, including maternal (eg, transplacental antibodies), heritable (eg, genes determining histo-blood group antigen structure), and environmental (eg, enteric pathogen exposure) factors [4]. The presence of asymptomatic enteric viruses—particularly nonpolio enteroviruses (NPEVs)—has consistently been linked with a reduction in the immunogenicity of oral poliovirus vaccine (OPV) [5], potentially reflecting competition between viruses at the cellular level [6], activation of innate antiviral immune pathways that inhibit OPV replication, or changes in lymphocyte responsiveness to poliovirus antigen. Notably, >100 enterovirus serotypes are known to infect humans. These can be separated into 4 distinct species (A–D) based on the sequence of the VP1 gene (a major determinant of antigenicity), of which polioviruses fall within species C [7, 8]. However, the relative inhibitory effect of different NPEV species or serotypes has not been investigated in any detail.

Other components of the gut microbiota may also be pertinent to OPV outcome. Enteric viruses seem to exploit signals from the bacterial microbiota when colonizing the intestinal mucosa [9]. Among infants in Bangladesh, the abundance of *Bifidobacterium* at the time of OPV administration correlated with poliovirus-specific immunoglobulin G and CD4+ T-cell responses [10]. However, the potential influence of microbiota composition on OPV replication and neutralizing antibody levels remains uncertain.

During a recent clinical trial in India, the presence of enteric viruses (of which the majority were NPEVs) was associated with reduced seroconversion to monovalent type 3 OPV (mOPV3) [11]. Here, we report on a follow-up study in which we tested whether OPV response was associated with specific enterovirus serotypes or species, short-term changes in enteric virus burden, or bacterial microbiota composition.

## METHODS

### Study Design

Between 5 August 2014 and 21 March 2015, we carried out a randomized, placebo-controlled trial evaluating the effect of azithromycin on the immunogenicity of mOPV3 among 6–11-month-old Indian infants (CTRI/2014/05/004588). The protocol and primary outcomes of this study have been published elsewhere [11]. Briefly, 754 infants lacking serum neutralizing antibodies to type 3 poliovirus were randomized 1:1 to receive a 3-day course of oral azithromycin (administered once daily at a dose of 10 mg/kg) or placebo, starting 14 days before the administration of a single dose of mOPV3. The study was approved by the institutional review board of the Christian Medical College, Vellore, India, and good clinical practice guidelines were followed throughout.

### Laboratory Testing and Bioinformatics

#### Enteropathogen Detection

**Figure 1.**
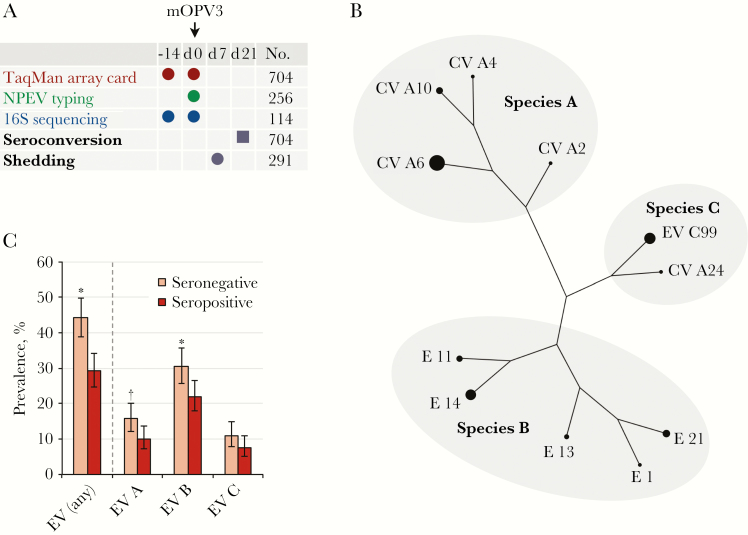
Association between nonpolio enteroviruses (NPEVs) and seroconversion. *A,* Study design. Circles represent stool samples; square, serum sample. “No.” column lists numbers of per-protocol infants. *B,* Enterovirus (EV) serotypes with a prevalence of ≥1%. The diameter of the circle at each branch tip is proportional to serotype prevalence. Phylogenetic relationships are as described by Oberste and colleagues [7]. Branch lengths are not proportional to phylogenetic distance. *C,* Enterovirus prevalence by seroconversion status. Error bars indicate 95% confidence intervals. *P < .01; †*P* < .05. Abbreviations: CV, coxsackievirus; E, echovirus; mOPV3, monovalent type 3 oral poliovirus vaccine.

Stool samples were collected from all infants before treatment (day −14) and on the day of vaccination (day 0) and tested for the presence of 40 different enteric bacterial, viral, and eukaryotic targets using TaqMan array cards (TACs), as described elsewhere (Figure 1A) [11]. A cycle threshold (Ct) value of 30 was used as a universal cutoff for pathogen detection. In all day 0 samples that were positive for enteroviruses in the TAC assay (targeting the 5’ untranslated region), we carried out a semi-nested polymerase chain reaction (PCR) assay targeting the VP1 gene [12]. Sanger sequencing of the amplified products was performed with a 3730XL DNA Analyzer, and enterovirus serotypes were assigned using the RIVM Enterovirus Genotyping Tool (version 1.0) [13].

#### Characterization of the Bacterial Microbiota

We assessed bacterial microbiota composition in 120 infants (60 per study arm) selected at random from the first 300 trial participants. Our laboratory and bioinformatic methods for microbiota assessment in this cohort have been published elsewhere [14]. For each infant, we included samples collected before treatment (day −14) and before vaccination (day 0). We also assessed samples from 40 adults living with trial participants to provide community-specific mature microbiota profiles (used as a reference for calculating microbiota age). After amplification and sequencing of the 16S ribosomal RNA gene V4 region with the Illumina MiSeq system, reads were assembled with the FLASH (Fast Length Adjustment of SHort reads) CCB, John Hopkins University computational tool, clustered de novo into operational taxonomic units (OTUs) using the uclust algorithm (implemented with MacQIIME software; version 1.8.0), and taxonomically assigned using the RDP classifier.

#### OPV Outcome

Serum samples collected 21 days after mOPV3 administration were tested for neutralizing antibodies against type 3 poliovirus, using a modified microneutralization assay with 2-fold serial dilutions ranging from 1:4 to 1:512 [15]. Seroconversion was defined as the detection of neutralizing antibodies at a dilution of ≥1:8. In a subset of 300 infants, shedding of type 3 poliovirus was assessed using real-time PCR in stool samples collected 7 days after vaccination [16].

### Statistical Analysis

#### Enteropathogen Burden

We included all per-protocol infants with available day 0 TAC data in the final analysis (n = 704). The association between each enterovirus species and OPV response (seroconversion or shedding as categorical dependent variables) was assessed by means of logistic regression. Age and study arm were included as covariates to account for the potential confounding of these variables with viral infection status and OPV outcome. We fitted univariate models for each species in turn and multivariate models that included all 3 species. Separate models were fitted for individual serotypes present in ≥1% of infants. The Fisher exact test was used to compare the prevalence of each enterovirus species by study arm. To examine whether mixed infections had an additive effect on OPV immunogenicity, we assessed the effect of enterovirus species count (as a categorical variable) on the odds of seroconversion. For each species in turn, we also compared the likelihood of heterospecific coinfection (≥1 enterovirus of another species) in infected versus uninfected infants, using the Fisher exact test.

To test whether the association between infection and OPV response varied across enterovirus species, we used the likelihood ratio test (LRT) to compare the fit of logistic regression models that only included presence or absence of any enterovirus with models that included enterovirus species as a categorical variable. Where multiple species were detected, infections were classified as “mixed,” and untypeable infections were classified as “unassigned.”

To examine whether changes in the burden of viral TAC targets (adenovirus, astrovirus, enterovirus, norovirus, rotavirus, and sapovirus) in the 14 days before immunization influenced OPV outcome, we classified viruses as absent (not detected on days −14 or 0), resolved (present on day −14, absent on day 0), recently acquired (absent on day −14, present on day 0), or persistent (present on days −14 and 0); these categories are hereafter referred to as “infection subclasses.” The impact of infection subclasses on OPV response was assessed by means of logistic regression, with age and study arm included as covariates. Infection subclasses were included if they contained ≥10 infants. We directly compared OPV outcome between infants with recently acquired versus persistent infections using the Fisher exact test; if a significant discrepancy was observed, we assessed whether this was associated with a difference in pathogen abundance (based on TAC Ct value), using the Wilcoxon rank sum test. Analyses were implemented in the programming language R [17], and associations with a *P* value <.05 were considered statistically significant.

#### Microbiota Composition

Among per-protocol infants in the microbiota subset, we compared baseline health and demographic characteristics by seroconversion status, using the Wilcoxon rank sum test (for continuous variables) or the Fisher exact test (for categorical variables). We obtained a minimum of 7708 sequences per sample after bioinformatic processing. To standardize sequencing depth, we performed analyses using 7500 sequences per sample. Microbiota measures of interest included alpha diversity, beta diversity, microbiota stability, microbiota age, and relative taxon abundance. These were compared according to OPV outcome, using the Wilcoxon rank sum test (for comparisons of taxon abundance), linear regression (for alpha diversity, microbiota stability, and microbiota age), or the adonis function (permutation-based analysis of variance; for beta diversity), with adjustment for infant age and study arm where possible. To account for multiple comparisons, *P* values for relative abundance comparisons were adjusted by Benjamini-Hochberg false discovery rate correction [18], applied separately at each taxonomic rank. The association between alpha diversity and enterovirus infection subclass was assessed using linear regression. We also used the machine-learning Random Forest algorithm to predict OPV outcome based on OTU relative abundance. For each model, we carried out 20 cycles of 10-fold cross-validation using the R package crossval, with 5000 trees per forest. Out-of-bag error rates and variable importance scores (based on Gini impurity) were determined across each of the 200 iterations of cross-validation. Sensitivity analyses of alpha and beta diversity were carried out, in which azithromycin recipients were excluded.

#### Data Availability

The 16S ribosomal RNA sequences have been deposited in the European Nucleotide Archive (accession No. PRJEB20773). An OTU table and relevant metadata have been published elsewhere [14].

## RESULTS

### NPEV Burden

Enteroviruses were present at the time of vaccination (day 0) in 107 of 367 infants (29%) who seroconverted and 149 of 337 (44%) who did not (odds ratio [OR], 0.49; 95% confidence interval [CI], .35–.67). Based on partial sequencing of the VP1 gene, species-level assignments were made in 247 (96%) of the 256 enterovirus-positive samples and serotype-level assignments were made in 234 (91%). Overall, we detected 56 NPEV serotypes, of which 11 were present in ≥1% of the study population (Figure 1B). Enteroviruses of species A, B, and C were detected in 90 (13%), 184 (26%), and 65 (9%) of the infants, respectively. Viruses in species D were absent. The receipt of azithromycin did not significantly affect the prevalence of any enterovirus species (Supplementary Figure 1). Infection with multiple enterovirus species was common; 76 of 704 infants (11%) harbored 2 species, and 8 of 704 (1%) were harbored 3 (Supplementary Figure 2). Moreover, the detection of any single enterovirus species was a significant risk factor for the presence of another (Table 1).

**Table 1.** Co-occurrence of Enterovirus Species

Enterovirus Subset	Prevalence of ≥1 Heterotypic Enterovirus, Number of Infants/Total (%)^a^
Species A positive	59/90 (65.6)
Species A negative	157/614 (25.6)
Species B positive	65/184 (35.3)
Species B negative	63/520 (12.1)
Species C positive	52/65 (80.0)
Species C negative	182/639 (28.5)

^a^ *P* < .001 for all comparisons (Fisher exact test).

### Association Between NPEVs and OPV Response

A negative association between NPEV detection and seroconversion to type 3 OPV was observed for each enterovirus species (OR [95% CI], 0.59 [.37–.93], 0.62 [.44–.87], and 0.67 [.40–1.13] for species A, B, and C, respectively, after adjustment for age and study arm) (Figure 1C). Although this effect was significant only for species A and B, there was no evidence that the effect size differed significantly between species (LRT, *P* = .19). The ORs were generally consistent in a multivariate model that included all species (Supplementary Table 1), and the presence of multiple species did not have an additive inhibitory effect on seroconversion (OR [95% CI], 0.45 [.31–.65], 0.67 [.41–1.09], and 0.43 [.09–1.80] for infants infected with 1, 2, or 3 species, respectively). With the exception of echovirus 14, associations between individual serotypes and seroconversion were not significant (Supplementary Table 2). Consistent with the data for seroconversion status, the association between concurrent NPEVs and postvaccination shedding did not vary significantly by species (LRT, *P* = .22) (Supplementary Table 1).

### Recently Acquired, Persistent, and Resolved Viruses

To investigate the relationship between recent changes in viral infection status and OPV response, we classified viruses as recently acquired, persistent, or resolved (Table 2). Recently acquired enteroviruses were the only infection subclass to be significantly associated with OPV response (OR, 0.38; 95% CI, .25–.59; *P* < .001). Notably, the seroconversion rate was significantly lower among infants with recently acquired as opposed to persistent enteroviruses (44 of 127 infants [35%] vs 63 of 129 [49%]; Fisher test, *P* = .02). The Ct values for persistent versus recently acquired enterovirus infections on day 0 did not differ significantly (mean Ct [standard deviation], 27.0 [2.2] vs 27.2 [2.1]; Wilcoxon rank sum test, *P* = .48), suggesting that the difference in immunogenicity between these groups was not driven by viral copy number.

**Table 2.** Association Between Viral Infection Subclasses and OPV3 Seroconversion^a^

Infection Status by Pathogen	Infants, No.	Seropositive Infants, No. (%)	OR (95% CI)	*P* Value	*P* Value (Recently Acquired vs Persistent)
Adenovirus					
Absent	507	269 (53.1)	…	…	…
Resolved	74	45 (60.8)	1.36 (.83–2.27)	.23	…
Recently acquired	88	40 (45.5)	0.73 (.46–1.16)	.19	…
Persistent	35	13 (37.1)	0.53 (.26–1.07)	.08	.43
Astrovirus					
Absent	684	355 (51.9)	…	…	…
Resolved	10	5 (50.0)	1.10 (.30–4.05)	.89	…
Recently acquired	10	7 (70.0)	2.30 (.58–10.81)	.24	…
Persistent	0	…	…	…	…
Enterovirus					
Absent	313	178 (56.9)	…	…	…
Resolved	135	82 (60.7)	1.24 (.82–1.90)	.30	…
Recently acquired	127	44 (34.6)	0.38 (.25–.59)	<.001	…
Persistent	129	63 (48.8)	0.70 (.46–1.06)	.09	.02
Norovirus					
Absent	604	318 (52.6)	…	…	…
Resolved	39	22 (56.4)	1.18 (.61–2.32)	.62	…
Recently acquired	46	20 (43.5)	0.70 (.38–1.28)	.25	…
Persistent	15	7 (46.7)	0.67 (.23–1.89)	.44	>.99
Rotavirus					
Absent	685	362 (52.8)	…	…	…
Resolved	6	2 (33.3)	…	…	…
Recently acquired	12	3 (25.0)	0.34 (.07–1.16)	.11	…
Persistent	1	0 (0.0)	…	…	…
Sapovirus					
Absent	671	348 (51.9)	…	…	…
Resolved	15	8 (53.3)	1.11 (.39–3.23)	.84	…
Recently acquired	16	9 (56.2)	1.30 (.47–3.7)	.62	…
Persistent	2	2 (100.0)	…	…	…

Abbreviations: CI, confidence interval; OPV3, type 3 oral poliovirus vaccine; OR, odds ratio.

^a^Age and study arm were included as covariates in all logistic regression models.

The odds of OPV shedding were reduced in infants with either recently acquired or persistent enteroviruses on day 0 (OR [95% CI], 0.55 [.28–1.08] and 0.47 [.25–.91], respectively). In contrast to the immunogenicity data, shedding rates did not differ significantly between these groups (25 of 53 infants [47%] vs 25 of 58 [43%] for recently acquired vs persistent enteroviruses; Fisher test, *P* > .99). No other viral infection subclasses were significantly associated with vaccine virus shedding (Supplementary Table 3).

### Association Between Bacterial Microbiota Composition and OPV Response

Of the 120 infants included in the microbiota subset, 114 (95%) completed the study per protocol. The effects of azithromycin on microbiota composition in these infants, including a reduction in the abundance of Proteobacteria and Verrucomicrobia, have been reported elsewhere [14]. Baseline health and socio-demographic characteristics were generally comparable between OPV responders and nonresponders in the microbiota subset (Supplementary Table 4), although failure to seroconvert was associated with a lower height-for-age *z* score at enrollment (day −14)—a discrepancy not apparent in the trial population as a whole [11].

**Figure 2.**
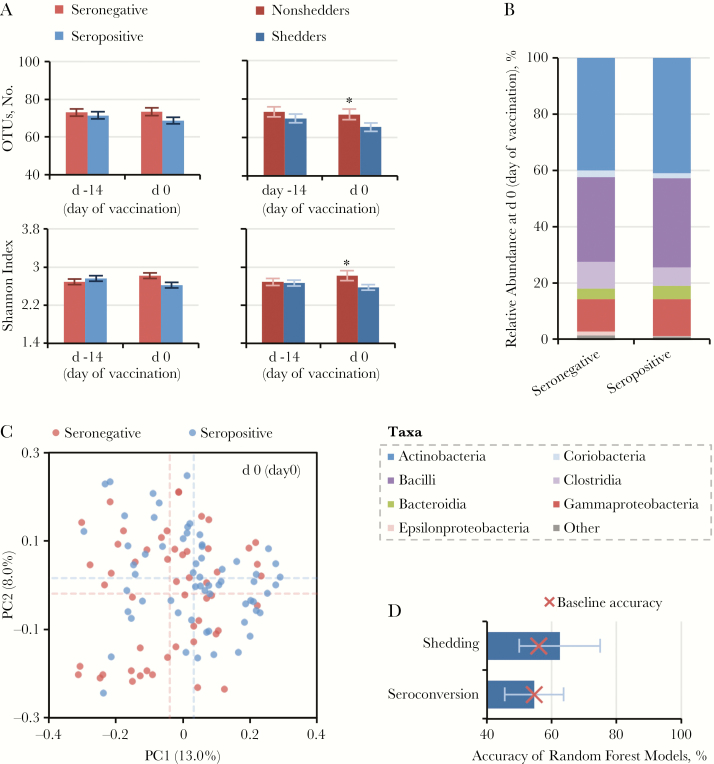
Association between microbiota composition on day of vaccination (day 0) and seroconversion. *A,* Operational taxonomic unit (OTU) count and Shannon index (presented as means with standard errors). *B,* Class-level composition of the bacterial microbiota. *C,* Unweighted UniFrac distances between samples, visualized using principal coordinate analysis. Mean values for each principal coordinate are indicated by dotted lines. *D,* Cross-validation accuracy of Random Forest models based on OTU abundance data (medians with interquartile ranges). The baseline accuracy is the expected accuracy if all individuals are assigned to the majority class. *P < .05; Abbreviations: PC, principal coordinate 1. Note- d 0 (day of vaccination) , d -14 (day of starting azithromycin or placebo)

We did not observe a strong correlation between composition of the bacterial microbiota at the time of vaccination (day 0) and OPV immunogenicity (Figure 2 and Table 3). UniFrac distance from adults (an indicator of microbiota age) was lower in nonresponders compared with responders (Table 3), whereas seroconversion status accounted for a significant but small proportion of variance among samples based on UniFrac distances (adonis function, *P* = .03; *R*2 = 0.013) (Table 3 and Figure 2C). We did not observe significant differences in OTU count, Shannon index, microbiota stability, or relative taxon abundances according to seroconversion status (Table 3 and Figure 2B). Random Forest models based on OTU abundance data failed to accurately distinguish infants according to OPV outcome (Figure 2D).

Similar results were obtained when comparing individuals according to vaccine shedding status (Table 3). In contrast to the immunogenicity data, however, OTU count and Shannon index were significantly higher among nonshedders than shedders, and the class Clostridia was enriched in nonshedders (mean relative abundance [standard deviation], 11.6% [10.2%] vs 5.65 [7.7%]; Wilcoxon rank sum test corrected for false discovery rate, *P= .*04) (Supplementary Table 5). Neither the OTU count nor the Shannon index varied significantly according to enterovirus infection subclass (LRT, *P* > .05) (Supplementary Figure 3), suggesting that the observed discrepancies in alpha diversity according to OPV outcome were not related to NPEV infection.

**Table 3.** Association Between Bacterial Microbiota Comparison at the Time of Vaccination (Day 0) and Oral Poliovirus Vaccine Response

Comparison	Test	Age/Arm as Covariates	Seroconversion			Shedding		
			Seropositive (n = 62)^a^	Seronegative (n = 52)	*P* Value	Shedders (n = 42)	Nonshedders (n = 33)	*P* Value
Alpha diversity								
OTU count, mean (SD)	LR	Yes	68.8 (14.1)	73.4 (15.3)	.20	65.4 (14.5)	72.0 (16.4)	.03
Shannon index, mean (SD)	LR	Yes	2.62 (0.47)	2.82 (0.55)	.08	2.57 (0.50)	2.84 (0.59)	.03
Beta diversity								
UniFrac distance between samples	Adonis function	Yes	*R* ^2^ = 0.013		.04	*R* ^2^ = 0.025		.007
Microbiota age, mean (SD), UniFrac distance from samples collected from noncohabiting adults	LR	Yes	0.833 (0.042)	0.811 (0.050)	.01	0.838 (0.046)	0.806 (0.052)	.001
Microbiota stability, mean (SD), UniFrac distance between d 0 and d–14	LR	Yes	0.470 (0.071)	0.460 (0.085)	.70	0.476 (0.078)	0.455 (0.088)	.20
Taxon abundance: phylum-, class-, genus-, and OTU-level relative abundance	WRS	No^b^	No discrepancies with FDR *P* < .15			Clostridia enriched in nonshedders^c^		
Cross-validation accuracy for Random Forest algorithm, median (IQR; baseline^d^), %	…	No^b^	54.5 (45.5–63.6; 54.5)			62.5 (50.0–75.0; 56.0)		

Abbreviations: FDR, adjusted for false discovery rate; IQR, interquartile range; LR, linear regression; OTUs, operational taxonomic units; SD, standard deviation; WRS, Wilcoxon rank sum test.

^a^A total of 120 infants were included in the microbiota subset, of whom 114 completed the study per protocol and were included in the final analyses.

^b^Although age and study arm could be included as covariates when applying linear regression or the adonis function, it was not possible to adjust for these variables when applying the WRS test (a nonparametric test) or the Random Forest algorithm.

^c^See Supplementary Table 5 for full results.

^d^Expected accuracy if all individuals are assigned to the majority class.

We observed no significant differences in microbiota composition according to OPV outcome in baseline (day −14) samples (Supplementary Table 6). Moreover, the primary outcomes were largely unaffected when infants in the azithromycin arm were excluded (Supplementary Table 6).

## DISCUSSION

The notion that concurrent enteroviruses may interfere with OPV immunogenicity dates back to the earliest trials of this vaccine [5]. Aside from a handful of relatively small association studies [19, 20], however, the extent to which this effect varies among enterovirus species or serotypes has not been tested. In a large cohort of Indian infants who received a single dose of mOPV3, we observed a significant inhibitory effect of concurrent NPEVs on OPV response. Moreover, we found this effect to be consistent across enterovirus species and enhanced when infection with these viruses was recently acquired.

The current study is among the first to characterize NPEV burden in asymptomatic infants living in a low- or middle-income country. Our findings highlight not only the high prevalence of NPEVs in this setting (35%) but also their considerable diversity. We observed a total of 56 different NPEV serotypes, of which coxsackievirus A6—one of the major serotypes implicated in clinical conditions such as hand, foot, and mouth disease [21]—was the most common. Two or more enterovirus species were detected in 11% of infants, and the presence of any single species was associated with an increased likelihood of heterospecific coinfection, suggesting that shared risk factors (eg, poor sanitation and hygiene) prompt multiple NPEV exposures. Several previous studies have used cell culture to characterize the burden of NPEVs among children presenting with acute flaccid paralysis. For example, NPEVs were documented in 15% of cases in West Africa [22] and 19% in southwestern India [23]. The higher prevalence of NPEVs reported here among asymptomatic infants may reflect the enhanced sensitivity of PCR-based versus culture-based detection methods [24].

Of the 56 NPEV serotypes observed in this population, 45 were present in <1% of infants, limiting our capacity to compare the effect of individual serotypes on OPV. The consistent species-level associations suggest that evolutionary (and antigenic) distance from poliovirus—a species C enterovirus—does not influence the outcomes of concurrent infection. Likewise, in cell culture, numerous NPEV serotypes have been shown to interfere with poliovirus replication [6]. Notably, enteroviruses exhibit long-term cycles in transmission that vary across serotypes; whereas some strains show regular annual peaks, others cause epidemics once every few years [25]. Our study was performed between August and March—a period typically associated with reduced NPEV transmission across India [26]. It is possible that distinct serotype-specific effects on OPV will only be apparent in particular seasons or years. Crucially, our findings are consistent with reports of seasonal variation in OPV immunogenicity [26] and suggest that planning OPV campaigns at times of year when enterovirus transmission is low may improve vaccine response.

By considering the viral infection burden at multiple time points before vaccination, we observed a reduction in OPV immunogenicity in infants with newly acquired as opposed to persistent enterovirus infections. This finding should be interpreted with caution, given its modest effect size and the fact that we did not find this discrepancy when considering OPV shedding. Nonetheless, it is plausible that recently acquired viruses may have an enhanced inhibitory effect on OPV response. Viral infections initiate a cascade of innate immune effectors that typically peak within several days of exposure [27]. Meanwhile, enteroviruses interfere with protein synthesis and secretion pathways to repress host interferon responses [28]. If the induction of innate antiviral immunity is responsible for the inhibitory effect of enteroviruses on OPV, the greater effect of recently acquired NPEVs compared with persistent infections could be due to the waning and/or repression of innate antiviral responses over time.

Intestinal viruses other than enteroviruses may also interfere with OPV. During the primary analysis of this cohort, rotaviruses and adenoviruses were associated with a reduction in OPV immunogenicity [11]. The same trends were evident here; however, the statistical power of individual comparisons was reduced by the distinction between persistent and recently acquired infection subclasses. Despite the potential inhibitory effect of other viruses, the considerably higher prevalence of enteroviruses ensures that this genus is likely to account for a much greater fraction of OPV failure.

In addition to the effect of intestinal viruses, dysbiosis of the bacterial microbiota has been proposed as a possible cause of impaired oral vaccine performance in low- or middle-income countries [29, 30]. Although lacking a consistent definition, dysbiosis has typically been linked with a reduction in microbiota diversity and a detectable shift (or “perturbation”) in overall composition [31]. In the current study, microbiota diversity was negatively correlated with OPV shedding. Given the modest effect size of the observed discrepancies and the fact that several previous studies have observed no such correlation [10, 32, 33], it seems unlikely that increased microbiota diversity represents a significant risk factor for vaccine failure, although it may be a marker for exposure to enteric infections. Other discrepancies in microbiota composition with respect to OPV response were modest, as exemplified by the inability of the Random Forest algorithm to accurately distinguish responders from nonresponders based on OTU abundance data. Thus, nonresponders did not exhibit any clear manifestations of microbiota dysbiosis.

Several other studies have looked at the possible effect of the bacterial microbiota on oral vaccine outcome. Among infants in Bangladesh, OPV response was positively correlated with *Bifidobacterium* abundance [10]. In Ghana, oral rotavirus vaccine immunogenicity was associated with an increased abundance of *Streptococcus bovis* and decreased abundance of the phylum Bacteroidetes [32], although no such discrepancies were apparent during a rotavirus vaccine study in Vellore, India [33]. In the current study, we did not identify any OTUs or genera that were significantly associated with OPV response. Although abundance of the class Clostridia was negatively correlated with vaccine shedding, given that older infants were less likely to respond to OPV [11], the observed discrepancy probably reflects the confounding of Clostridia abundance with age [14]. Differences in infant age, vaccine, laboratory methods, trial setting, and immunogenicity measure may all have contributed to the discrepancies in findings among studies reporting on the association between bacterial microbiota composition and oral vaccine response. For now, reproducible signatures of oral vaccine failure within the bacterial microbiota remain elusive.

Our study has several limitations. Biases in amplification efficiency may have undermined our characterization of the bacterial microbiota [34], and a focus on relative rather than absolute taxon abundance (accounting for variation in total microbial load) may have obscured potentially relevant associations [14, 35]. By applying a Ct cutoff of 30 for pathogen targets during the TAC assays, we may have failed to characterize the effect of enteric viruses present at low abundance. Finally, during our analysis of infection subclasses, it is possible that the replacement of one virus with another would be mistakenly categorized as a persistent infection, because we did not determine enterovirus serotype in samples collected 14 days before vaccination.

In conclusion, we did not observe any signs of bacterial microbiota dysbiosis among infants in India who failed to respond to mOPV3. The presence of NPEVs was associated with a lower response to OPV that was consistent across enterovirus species, and recently acquired enteroviruses seemed to inhibit OPV immunogenicity more than persistent infections. Although these findings do not preclude a role for more entrenched risk factors of OPV failure, such as the chronic inflammation associated with environmental enteropathy, they suggest that the likelihood of responding to OPV may fluctuate from week to week and are consistent with seasonal trends in OPV immunogenicity that may reflect the abundance of NPEVs.

## Supplementary Material

Supplementary Figure S1

Supplementary Figure S2

Supplementary Figure S3

Supplementary Table S1

Supplementary Table S2

Supplementary Table S3

Supplementary Table S4

Supplementary Table S5

Supplementary Table S6

Supplementary Table S7

Supplementary Table S8

## Notes

***Author contributions.*** I. P., MD; N. C. G., D Phil and G. K., MD, PhD had full access to all the data in the study and had final responsibility for the decision to submit for publication. I. P., E. P. K. P., N. C. G., B. K., and G. K. conceived the study. S. P. K., J. H. P. and J. J. managed the clinical trial. I. P., E. P. K. P., S. G., D. J. A. , S. S., M. I-G., R. R., and G. K. led the laboratory work. I. P., E. P. K. P., and N. C. G. led the statistical analysis. All authors contributed to the interpretation of the data, writing of the report, and approved the final manuscript.

***Acknowledgments.*** We are grateful to Margarita Pons-Salort for providing valuable comments on the manuscript.

***Financial support.*** This study was funded by the Bill and Melinda Gates Foundation (grant OPP1039135 to N. C. G. and G. K.).

***Potential conflicts of interests.*** M. I. G. received support from an Institutional Strategic Support Fund Wellcome Trust grant awarded to the University of Liverpool and the Health Protection Research Unit in Gastrointestinal Infections, National Institute for Health Research at the University of Liverpool (grant NIHR HPRU 2012-10038). All other authors report no potential conflicts. All authors have submitted the ICMJE Form for Disclosure of Potential Conflicts of Interest. Conflicts that the editors consider relevant to the content of the manuscript have been disclosed.
