# Supplementary figures and images for: Influence of Nonpolio Enteroviruses and the Bacterial Gut Microbiota on Oral Poliovirus Vaccine Response: A Study from South India
Source: J Infect Dis. 2018 Sep 24;219(8):1178–86. doi: 10.1093/infdis/jiy568 (PMC6601701; doi:10.1093/infdis/jiy568)

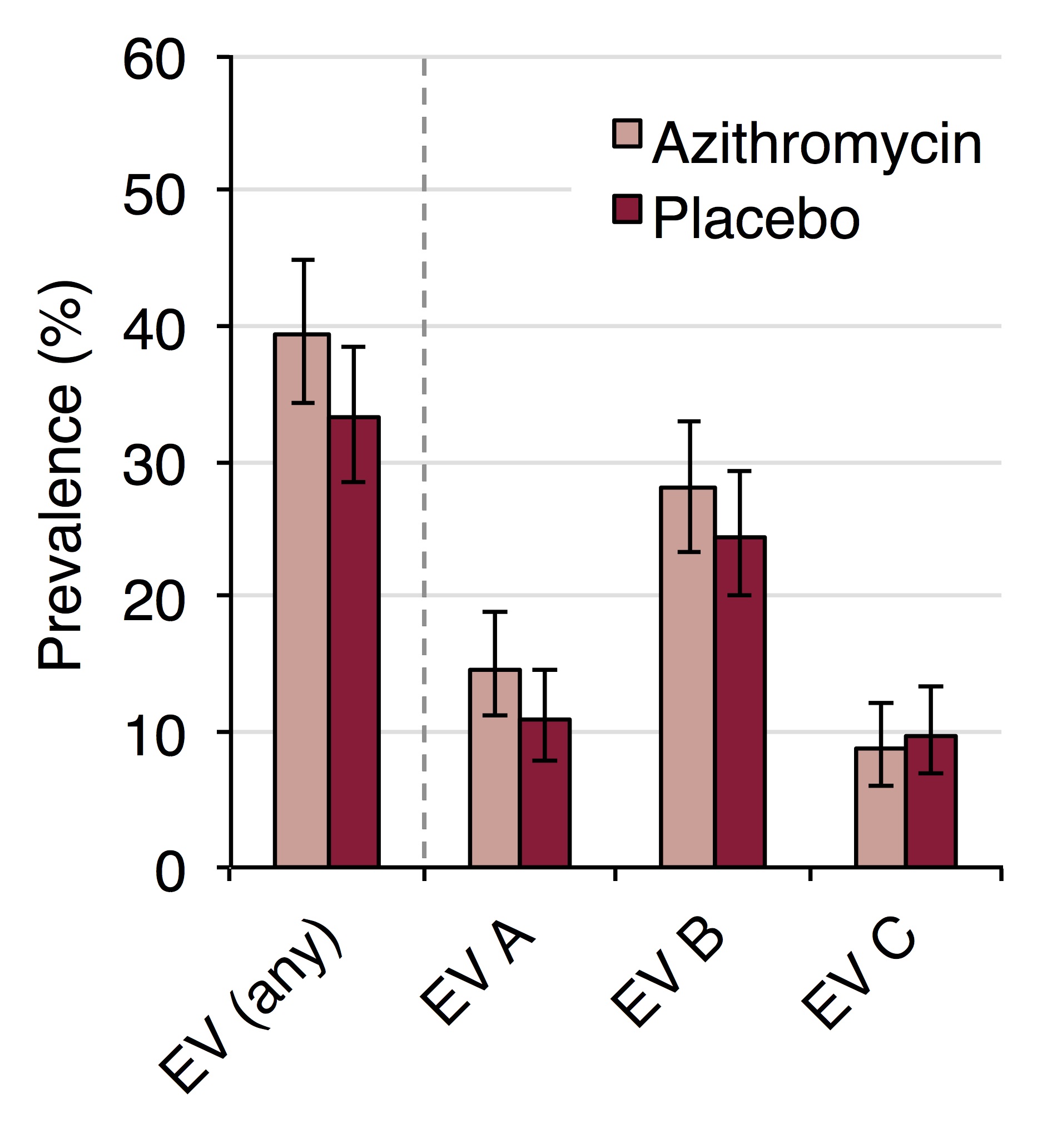

Supplement: Supplementary Figure S1 [file jiy568_suppl_supplementary_figure_s1.jpeg]

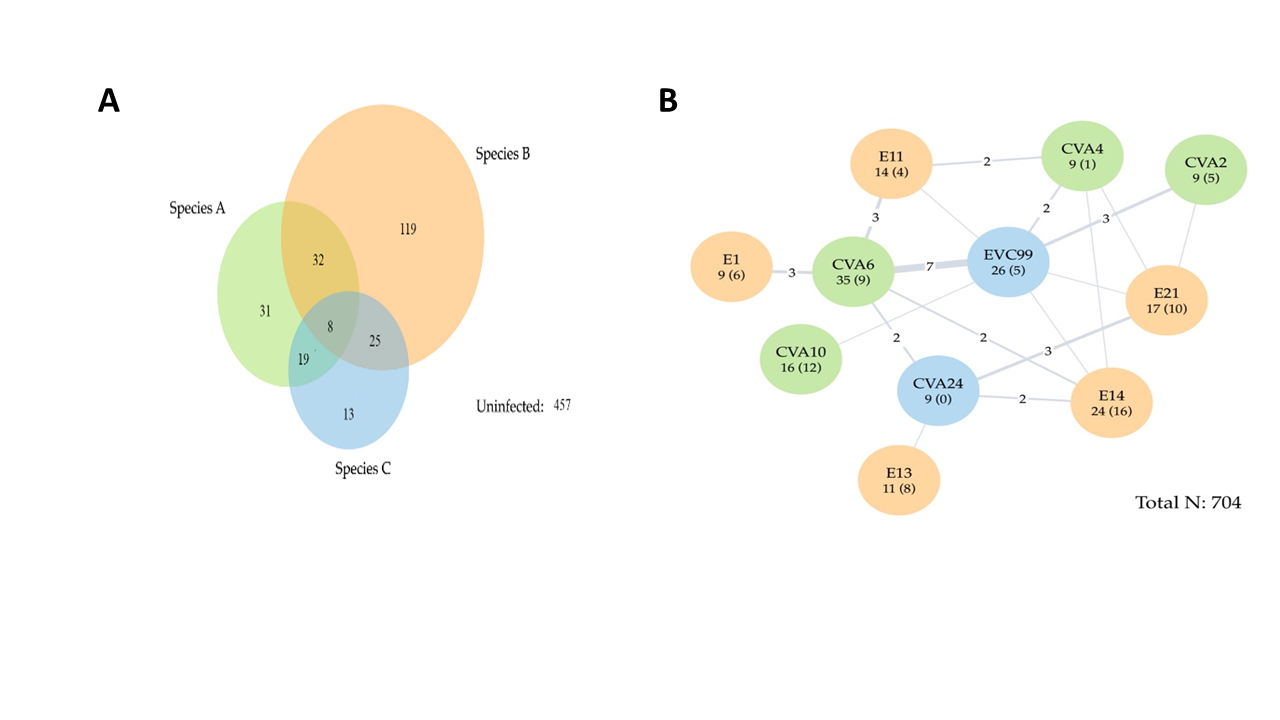

Supplement: Supplementary Figure S2 [file jiy568_suppl_supplementary_figure_s2.png]

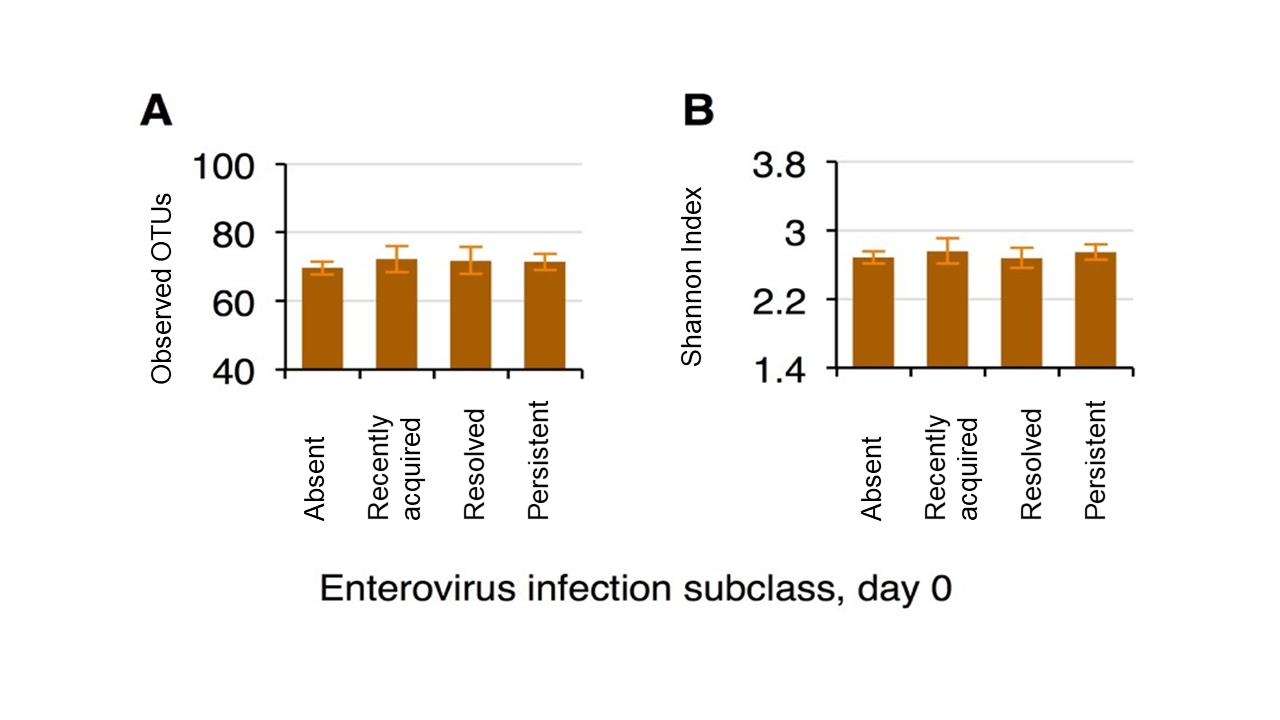

Supplement: Supplementary Figure S3 [file jiy568_suppl_supplementary_figure_s3.png]
