# Supplementary material for: Influence of Nonpolio Enteroviruses and the Bacterial Gut Microbiota on Oral Poliovirus Vaccine Response: A Study from South India
Source: J Infect Dis. 2018 Sep 24;219(8):1178–86. doi: 10.1093/infdis/jiy568 (PMC6601701; doi:10.1093/infdis/jiy568)
Supplement: Supplementary Table S1 [file jiy568_suppl_supplementary_table_s1.docx]

| **Table S1. Association between enterovirus species and oral poliovirus vaccine outcome: univariate versus multivariate logistic regression.** | | | | | |
| --- | --- | --- | --- | --- | --- |
|  |  | Univariate | | Multivariate | |
| Outcome | Species | OR (95% CI) | p | OR (95% CI) | p |
| Seroconversion | A | 0.59 (0.37–0.93) | 0.023 | 0.66 (0.41–1.07) | 0.091 |
|  | B | 0.62 (0.44–0.87) | 0.007 | 0.66 (0.47–0.94) | 0.021 |
|  | C | 0.67 (0.40–1.13) | 0.135 | 0.86 (0.49–1.48) | 0.581 |
| Shedding | A | 1.31 (0.61–2.89) | 0.486 | 1.55 (0.70–3.54) | 0.284 |
|  | B | 0.50 (0.29–0.84) | 0.010 | 0.51 (0.29–0.88) | 0.017 |
|  | C | 0.59 (0.24–1.36) | 0.216 | 0.74 (0.29–1.83) | 0.512 |
| Age and study arm were included as covariates in all logistic regression models. | | | | | |
