# Supplementary material for: Influence of Nonpolio Enteroviruses and the Bacterial Gut Microbiota on Oral Poliovirus Vaccine Response: A Study from South India
Source: J Infect Dis. 2018 Sep 24;219(8):1178–86. doi: 10.1093/infdis/jiy568 (PMC6601701; doi:10.1093/infdis/jiy568)
Supplement: Supplementary Table S2 [file jiy568_suppl_supplementary_table_s2.docx]

| **Table S2. Association between enterovirus serotypes and oral poliovirus vaccine outcome.** | | | | | | |  | |  | |  |
| --- | --- | --- | --- | --- | --- | --- | --- | --- | --- | --- | --- |
| Species | Serotype | Seropositive (n = 367) | Seronegative (n = 337) | OR (95% CI) | p | Shedders (n = 156) | | Non-shedders (n = 135) | |  |  |
| A | CV-A6 | 14 (3.8) | 21 (6.2) | 0.57 (0.28–1.13) | 0.111 | 4 (2.6) | | 5 (3.7) | |  |  |
|  | CV-A10 | 8 (2.2) | 8 (2.4) | 0.79 (0.28–2.17) | 0.636 | 8 (5.1) | | 1 (0.7) | |  |  |
|  | CV-A2 | 2 (0.5) | 7 (2.1) | 0.28 (0.04–1.17) | 0.112 | 0 (0.0) | | 3 (2.2) | |  |  |
|  | CV-A4 | 3 (0.8) | 6 (1.8) | 0.50 (0.10–1.95) | 0.338 | 0 (0.0) | | 1 (0.7) | |  |  |
|  | CV-A5 | 1 (0.3) | 4 (1.2) | - | - | 2 (1.3) | | 1 (0.7) | |  |  |
|  | EV-A76 | 3 (0.8) | 2 (0.6) | - | - | 2 (1.3) | | 1 (0.7) | |  |  |
|  | CV-A7 | 0 (0) | 1 (0.3) | - | - | 0 (0.0) | | 0 (0.0) | |  |  |
|  | CV-A8 | 1 (0.3) | 0 (0) | - | - | 0 (0.0) | | 0 (0.0) | |  |  |
|  | EV-A71 | 0 (0) | 1 (0.3) | - | - | 0 (0.0) | | 0 (0.0) | |  |  |
|  | EV-A90 | 1 (0.3) | 0 (0) | - | - | 1 (0.6) | | 0 (0.0) | |  |  |
|  | EV-A91 | 1 (0.3) | 0 (0) | - | - | 1 (0.6) | | 0 (0.0) | |  |  |
|  | Unassigned | 4 (1.1) | 3 (0.9) | - | - | 1 (0.6) | | 1 (0.7) | |  |  |
| B | E-14 | 6 (1.6) | 18 (5.3) | 0.29 (0.10–0.69) | 0.009 | 3 (1.9) | | 8 (6.0) | |  |  |
|  | E-21 | 5 (1.4) | 12 (3.6) | 0.36 (0.11–0.99) | 0.059 | 4 (2.6) | | 7 (5.2) | |  |  |
|  | E-11 | 7 (1.9) | 7 (2.1) | 1.01 (0.34–3.01) | 0.980 | 3 (1.9) | | 2 (1.5) | |  |  |
|  | E-13 | 5 (1.4) | 6 (1.8) | 0.75 (0.21–2.53) | 0.639 | 1 (0.6) | | 4 (3.0) | |  |  |
|  | E-1 | 7 (1.9) | 2 (0.6) | 3.20 (0.77–21.62) | 0.149 | 2 (1.3) | | 2 (1.5) | |  |  |
|  | E-33 | 3 (0.8) | 4 (1.2) | - | - | 2 (1.3) | | 4 (3.0) | |  |  |
|  | E-6 | 4 (1.1) | 3 (0.9) | - | - | 2 (1.3) | | 1 (0.7) | |  |  |
|  | E-7 | 3 (0.8) | 4 (1.2) | - | - | 3 (1.9) | | 0 (0.0) | |  |  |
|  | CV-B3 | 4 (1.1) | 2 (0.6) | - | - | 0 (0.0) | | 0 (0.0) | |  |  |
|  | E-29 | 3 (0.8) | 3 (0.9) | - | - | 2 (1.3) | | 1 (0.7) | |  |  |
|  | E-30 | 2 (0.5) | 4 (1.2) | - | - | 0 (0.0) | | 1 (0.7) | |  |  |
|  | E-12 | 2 (0.5) | 4 (1.2) | - | - | 2 (1.3) | | 3 (2.2) | |  |  |
|  | E-15 | 2 (0.5) | 3 (0.9) | - | - | 1 (0.6) | | 2 (1.5) | |  |  |
|  | E-24 | 4 (1.1) | 1 (0.3) | - | - | 2 (1.3) | | 1 (0.7) | |  |  |
|  | E-5 | 4 (1.1) | 1 (0.3) | - | - | 4 (2.6) | | 1 (0.7) | |  |  |
|  | EV-B80 | 1 (0.3) | 3 (0.9) | - | - | 0 (0.0) | | 0 (0.0) | |  |  |
|  | CV-A9 | 1 (0.3) | 2 (0.6) | - | - | 0 (0.0) | | 0 (0.0) | |  |  |
|  | EV-B82 | 2 (0.5) | 1 (0.3) | - | - | 1 (0.6) | | 1 (0.7) | |  |  |
|  | CV-B1 | 0 (0) | 2 (0.6) | - | - | 0 (0.0) | | 0 (0.0) | |  |  |
|  | E-20 | 1 (0.3) | 1 (0.3) | - | - | 0 (0.0) | | 1 (0.7) | |  |  |
|  | E-27 | 1 (0.3) | 1 (0.3) | - | - | 0 (0.0) | | 0 (0.0) | |  |  |
|  | EV-B73 | 0 (0) | 2 (0.6) | - | - | 0 (0.0) | | 1 (0.7) | |  |  |
|  | EV-B81 | 2 (0.5) | 0 (0) | - | - | 1 (0.6) | | 0 (0.0) | |  |  |
|  | EV-B86 | 1 (0.3) | 1 (0.3) | - | - | 1 (0.6) | | 0 (0.0) | |  |  |
|  | EV-B88 | 1 (0.3) | 1 (0.3) | - | - | 0 (0.0) | | 0 (0.0) | |  |  |
|  | CV-B6 | 0 (0) | 1 (0.3) | - | - | 0 (0.0) | | 0 (0.0) | |  |  |
|  | E-17 | 0 (0) | 1 (0.3) | - | - | 0 (0.0) | | 0 (0.0) | |  |  |
|  | E-18 | 0 (0) | 1 (0.3) | - | - | 0 (0.0) | | 1 (0.7) | |  |  |
|  | E-19 | 0 (0) | 1 (0.3) | - | - | 0 (0.0) | | 0 (0.0) | |  |  |
|  | E-2 | 1 (0.3) | 0 (0) | - | - | 1 (0.6) | | 0 (0.0) | |  |  |
|  | E-25 | 0 (0) | 1 (0.3) | - | - | 1 (0.6) | | 0 (0.0) | |  |  |
|  | E-26 | 0 (0) | 1 (0.3) | - | - | 0 (0.0) | | 0 (0.0) | |  |  |
|  | E-3 | 1 (0.3) | 0 (0) | - | - | 0 (0.0) | | 0 (0.0) | |  |  |
|  | E-31 | 0 (0) | 1 (0.3) | - | - | 0 (0.0) | | 1 (0.7) | |  |  |
|  | E-32 | 0 (0) | 1 (0.3) | - | - | 0 (0.0) | | 0 (0.0) | |  |  |
|  | EV-B100 | 0 (0) | 1 (0.3) | - | - | 0 (0.0) | | 0 (0.0) | |  |  |
|  | EV-B101 | 1 (0.3) | 0 (0) | - | - | 1 (0.6) | | 0 (0.0) | |  |  |
|  | EV-B75 | 0 (0) | 1 (0.3) | - | - | 0 (0.0) | | 0 (0.0) | |  |  |
|  | EV-B93 | 1 (0.3) | 0 (0) | - | - | 0 (0.0) | | 0 (0.0) | |  |  |
|  | Unassigned | 8 (2.2) | 7 (2.1) | 1.04 (0.37–3.02) | 0.941 | 1 (0.6) | | 6 (4.5) | |  |  |
| C | EV-C99 | 13 (3.5) | 13 (3.9) | 0.94 (0.43-2.09) | 0.885 | 2 (1.3) | | 4 (3.0) | |  |  |
|  | CV-A24 | 3 (0.8) | 6 (1.8) | 0.44 (0.09-1.7) | 0.253 | 3 (1.9) | | 5 (3.7) | |  |  |
|  | CV-A1 | 3 (0.8) | 4 (1.2) | - | - | 0 (0.0) | | 1 (0.7) | |  |  |
|  | CV-A19 | 3 (0.8) | 1 (0.3) | - | - | 2 (1.3) | | 2 (1.5) | |  |  |
|  | CV-A17 | 2 (0.5) | 1 (0.3) | - | - | 0 (0.0) | | 0 (0.0) | |  |  |
|  | EV-C116 | 0 (0) | 1 (0.3) | - | - | 0 (0.0) | | 1 (0.7) | |  |  |
|  | PV-3 | 0 (0) | 1 (0.3) | - | - | 0 (0.0) | | 1 (0.7) | |  |  |
|  | Unassigned | 4 (1.1) | 10 (3) | 0.38 (0.1-1.16) | 0.106 | 3 (1.9) | | 1 (0.7) | |  |  |
| Unclassified | | 3 (0.8) | 6 (1.8) | 0.40 (0.08–1.54) | 0.201 | 2 (1.3 | | 3 (2.2) | |  |  |
| Data are n (%). For enteroviruses present in at least 1% of the population, the association between viral detection and seroconversion was determined via logistic regression, with age included as a covariate. Enterovirus prevalence and post-vaccination shedding data are presented for only the subset of infants for which both results available. CI, confidence interval; CV, coxsackievirus; E, echovirus; EV, enterovirus; OR, odds ratio; PV, poliovirus. | | | | | | | | | | | |
