# Supplementary material for: Influence of Nonpolio Enteroviruses and the Bacterial Gut Microbiota on Oral Poliovirus Vaccine Response: A Study from South India
Source: J Infect Dis. 2018 Sep 24;219(8):1178–86. doi: 10.1093/infdis/jiy568 (PMC6601701; doi:10.1093/infdis/jiy568)
Supplement: Supplementary Table S3 [file jiy568_suppl_supplementary_table_s3.docx]

| **Table S3. Association between resolved, persistent, and recently acquired viral infections and vaccine shedding.** | | | | | | | |
| --- | --- | --- | --- | --- | --- | --- | --- |
| Pathogen | Infection status | | N | Shedders,  n (%) | OR (95% CI) | p | p ^Rec vs Pe^ |
| Adenovirus | | Absent | 202 | 108 (53.5) |  |  |  |
|  | | Resolved | 30 | 22 (73.3) | 2.24 (0.97–5.62) | 0.068 |  |
|  | | Recently acquired | 39 | 16 (41.0) | 0.57 (0.28–1.14) | 0.116 |  |
|  | | Persistent | 20 | 10 (50.0) | 0.94 (0.36–2.42) | 0.892 | 1.000 |
| Enterovirus | | Absent | 114 | 68 (59.6) |  |  |  |
|  | | Resolved | 66 | 38 (57.6) | 1.04 (0.55–1.97) | 0.902 |  |
|  | | Recently acquired | 53 | 25 (47.2) | 0.55 (0.28–1.08) | 0.083 |  |
|  | | Persistent | 58 | 25 (43.1) | 0.49 (0.25–0.93) | 0.031 | 1.000 |
| Norovirus | | Absent | 263 | 140 (53.2) |  |  |  |
|  | | Resolved | 12 | 6 (50.0) | 0.81 (0.24–2.67) | 0.718 |  |
|  | | Recently acquired | 13 | 8 (61.5) | 1.22 (0.39–4.18) | 0.733 |  |
|  | | Persistent | 3 | 2 (66.7) | - | - | - |
| Age and study arm were included as covariates in all logistic regression models. The numbers of infants with resolved, recently acquired, or persistent infections for astrovirus, rotavirus, and sapovirus were insufficient for inclusion in these analyses (n < 10). Abbreviations: CI, confidence interval; OR, odds ratio; Rec, recently acquired; Pe, Persistent | | | | | | | |
