# Supplementary material for: Influence of Nonpolio Enteroviruses and the Bacterial Gut Microbiota on Oral Poliovirus Vaccine Response: A Study from South India
Source: J Infect Dis. 2018 Sep 24;219(8):1178–86. doi: 10.1093/infdis/jiy568 (PMC6601701; doi:10.1093/infdis/jiy568)
Supplement: Supplementary Table S4 [file jiy568_suppl_supplementary_table_s4.docx]

| **Table S4. Association between baseline characteristics and seroconversion after OPV among infants included in the microbiota subset.** | | | |
| --- | --- | --- | --- |
|  | Seropositive (n = 62) | Seronegative (n = 52) | p |
| Azithromycin arm | 33 (53.2) | 23 (44.2) | 0.354 |
| Age (months) | 7.9 (1.6) | 8.1 (1.7) | 0.390 |
| Female | 35 (56.5) | 25 (48.1) | 0.452 |
| Mother’s education |  |  | 0.728 |
| Illiterate | 1 (1.6) | 2 (3.8) | 0.728 |
| Primary (1–5 years) | 7 (11.3) | 6 (11.5) |  |
| Middle (6–8 years) | 16 (25.8) | 18 (34.6) |  |
| Secondary (9–12 years) | 32 (51.6) | 23 (44.2) |  |
| University graduate (13+ years) | 6 (9.7) | 3 (5.8) |  |
| House roof type |  |  | 0.318 |
| Concrete | 35 (56.5) | 22 (42.3) |  |
| Tiled | 11 (17.7) | 13 (25.0) |  |
| Thatched | 16 (25.8) | 17 (32.7) |  |
| Trivalent oral poliovirus vaccine doses | 4.5 (1.3) | 4.4 (1.2) | 0.731 |
| Health status |  |  |  |
| Diarrhoea in the 7 days before vaccination | 2 (3.2) | 1 (1.9) | 1.000 |
| Breastfed (yes vs no) | 3 (4.8) | 3 (5.8) | 1.000 |
| Height-for-age Z score | -0.6 (1.1) | -1.1 (1.1) | 0.029^a^ |
| Weight-for-age Z score | -1.1 (0.9) | -1.2 (0.9) | 0.481 |
| Data are mean (standard deviation) or n (%). Responders and non-responders were compared using Wilcoxon’s rank sum test or Fisher’s exact test. Six infants in the microbiota subset (three responders and three non-responders) did not complete the study per protocol and were excluded from the analysis. A comparison between baseline characteristics in the microbiota analysis subset and the primary trial cohort is provided in Table S7.  ^a^ No significant association between height-for-age *Z* score and seroconversion was observed in the per-protocol study population as a whole (n = 705). | | | |
