# Supplementary material for: Influence of Nonpolio Enteroviruses and the Bacterial Gut Microbiota on Oral Poliovirus Vaccine Response: A Study from South India
Source: J Infect Dis. 2018 Sep 24;219(8):1178–86. doi: 10.1093/infdis/jiy568 (PMC6601701; doi:10.1093/infdis/jiy568)
Supplement: Supplementary Table S5 [file jiy568_suppl_supplementary_table_s5.docx]

| **Table S5. Summary of taxon abundance comparisons according to type 3 poliovirus response** | | | | | | |
| --- | --- | --- | --- | --- | --- | --- |
| Taxonomic classification | Prevalence (%) | | Relative abundance  (mean % ± s.d.) | | p | FDR p |
| Seroconversion | sero+  (n = 62) | sero-  (n = 52) | sero+  (n = 62) | sero-  (n = 52) |  |  |
| **Phylum (all)** |  |  |  |  |  |  |
| Actinobacteria | 100.0 | 100.0 | 42.824 ± 18.644 | 42.455 ± 20.126 | 0.9660 | 0.9660 |
| Bacteroidetes | 80.6 | 94.2 | 4.821 ± 10.722 | 3.777 ± 6.498 | 0.0853 | 0.2985 |
| Firmicutes | 100.0 | 100.0 | 38.633 ± 19.072 | 39.858 ± 20.838 | 0.7848 | 0.9660 |
| Fusobacteria | 22.6 | 38.5 | 0.010 ± 0.027 | 0.958 ± 4.840 | 0.0457 | 0.2985 |
| Proteobacteria | 98.4 | 100.0 | 13.296 ± 16.585 | 12.854 ± 11.819 | 0.4493 | 0.7862 |
| Verrucomicrobia | 11.3 | 9.6 | 0.385 ± 2.976 | 0.070 ± 0.398 | 0.8517 | 0.9660 |
| Other | 83.9 | 75.0 | 0.032 ± 0.026 | 0.027 ± 0.031 | 0.1749 | 0.4081 |
| **Class (FDR p <0.15)** | | |  |  |  |  |
| NA | - | - | - | - | - | - |
| **Genus (FDR p <0.15)** | | |  |  |  |  |
| NA | - | - | - | - | - | - |
| **OTU (FDR p <0.15)** | | |  |  |  |  |
| NA | - | - | - | - | - | - |
| Shedding | shed+  (n = 42) | shed-  (n = 33) | shed+  (n = 42) | shed-  (n = 33) |  |  |
| **Phylum (all)** |  |  |  |  |  |  |
| Actinobacteria | 100.0 | 100.0 | 41.777 ± 20.422 | 44.672 ± 17.791 | 0.4226 | 0.5913 |
| Bacteroidetes | 78.6 | 100.0 | 4.383 ± 9.604 | 4.408 ± 7.486 | 0.0734 | 0.4600 |
| Firmicutes | 100.0 | 100.0 | 37.966 ± 21.086 | 39.233 ± 20.560 | 0.7462 | 0.7462 |
| Fusobacteria | 19.0 | 27.3 | 0.032 ± 0.150 | 0.009 ± 0.022 | 0.5068 | 0.5913 |
| Proteobacteria | 97.6 | 100.0 | 15.236 ± 18.064 | 11.557 ± 12.988 | 0.3615 | 0.5913 |
| Verrucomicrobia | 19.0 | 6.1 | 0.578 ± 3.614 | 0.096 ± 0.498 | 0.1314 | 0.4600 |
| Other | 81.0 | 75.8 | 0.028 ± 0.024 | 0.026 ± 0.032 | 0.4980 | 0.5913 |
| **Class (FDR p <0.15)** | | |  |  |  |  |
| Clostridia | 100.0 | 100.0 | 5.646 ± 7.668 | 11.558 ± 10.208 | 0.0031 | 0.0435 |
| **Genus (FDR p <0.15)** | | |  |  |  |  |
| NA | - | - | - | - | - | - |
| **OTU (FDR p <0.15)** | | |  |  |  |  |
| NA | - | - | - | - | - | - |
| Abbreviations: FDR, adjusted by Benjamini–Hochberg false discovery rate correction; OTU, 97%-identity operational taxonomic unit; sero+, responders; sero-, non-responders; shed+, shedders; shed-, non-shedders. | | | | | | |
