# Supplementary material for: Influence of Nonpolio Enteroviruses and the Bacterial Gut Microbiota on Oral Poliovirus Vaccine Response: A Study from South India
Source: J Infect Dis. 2018 Sep 24;219(8):1178–86. doi: 10.1093/infdis/jiy568 (PMC6601701; doi:10.1093/infdis/jiy568)
Supplement: Supplementary Table S6 [file jiy568_suppl_supplementary_table_s6.docx]

| **Table S6. Bacterial microbiota composition: baseline (day -14) comparisons and sensitivity analyses** | | | | | | | | |
| --- | --- | --- | --- | --- | --- | --- | --- | --- |
| Comparison | Day | Population | Seroconversion | | | Shedding | | |
|  |  |  | Positive | Negative | p | Positive | Negative | p |
| OTU count | -14 | Full | 71.5 ± 14.7 | 73.0 ± 14.3 | 0.892 | 69.9 ± 14.9 | 73.2 ± 15.1 | 0.357 |
|  | 0 | Placebo arm | 71.9 ± 13.7 | 75.2 ± 13.7 | 0.503 | 67.2 ± 13.6 | 76.4 ± 13.3 | 0.020 |
| Shannon index | -14 | Full | 2.76 ± 0.48 | 2.69 ± 0.49 | 0.196 | 2.66 ± 0.45 | 2.70 ± 0.44 | 0.799 |
|  | 0 | Placebo arm | 2.72 ± 0.52 | 2.86 ± 0.55 | 0.388 | 2.62 ± 0.54 | 2.96 ± 0.62 | 0.072 |
| Beta diversity | -14 | Full | R^2^ = 0.011 | | 0.152 | R^2^ = 0.015 | | 0.221 |
|  | 0 | Placebo arm | R^2^ = 0.021 | | 0.139 | R^2^ = 0.034 | | 0.142 |
| Microbiota age | -14 | Full | 0.825 ± 0.045 | 0.814 ± 0.047 | 0.644 | 0.827 ± 0.048 | 0.817 ± 0.051 | 0.349 |
| Taxon abundance | -14 | Full | No discrepancies with FDR p<0.15 | | | No discrepancies with FDR p<0.15 | | |
| Random Forests | -14 | Full | Median 54.5% (IQR 45.5–63.6%; baseline accuracy: 54.5%) | | | Median 57.1% (IQR 50.0–71.4%; baseline accuracy: 56.0%) | | |
| Data are mean ± standard deviation unless otherwise stated. See Table 3 for additional details of measures and statistical tests. FDR, adjusted for false discovery rate; IQR, interquartile range. | | | | | | | | |
