# Supplementary material for: Influence of Nonpolio Enteroviruses and the Bacterial Gut Microbiota on Oral Poliovirus Vaccine Response: A Study from South India
Source: J Infect Dis. 2018 Sep 24;219(8):1178–86. doi: 10.1093/infdis/jiy568 (PMC6601701; doi:10.1093/infdis/jiy568)
Supplement: Supplementary Table S7 [file jiy568_suppl_supplementary_table_s7.docx]

| **Table S7. Baseline characteristics in the per-protocol study population and the microbiota analysis subset.** | | |
| --- | --- | --- |
|  | Per-protocol study population  (n = 705) | Microbiota analysis subset  (n = 114) |
| Age (months) | 7.5 (1.5) | 8.0 (1.7) |
| Female | 374 (53.0) | 60 (52.6) |
| Height (length in cm) | 66.9 (3.2) | 67.7 (3.2) |
| Weight (in kg) | 7.3 (0.9) | 7.3 (0.9) |
| Height-for-age Z score | -1.2 (1.1) | -0.8 (1.1) |
| Weight-for-age Z score | -1.1 (1.0) | -1.1 (0.9) |
| Breastfed | 653 (92.6) | 108 (94.7) |
| Data are mean (standard deviation) or n (%). | | |
