# Supplementary material for: Influence of Nonpolio Enteroviruses and the Bacterial Gut Microbiota on Oral Poliovirus Vaccine Response: A Study from South India
Source: J Infect Dis. 2018 Sep 24;219(8):1178–86. doi: 10.1093/infdis/jiy568 (PMC6601701; doi:10.1093/infdis/jiy568)
Supplement: Supplementary Table S8 [file jiy568_suppl_supplementary_table_s8.docx]

**Table S8- Serotypes commonly present in enterovirus co-infections**

| **Enterovirus Serotype*** | **% co-infections present (N=84)** | **Common co-infecting enterovirus serotypes n(%)** |
| --- | --- | --- |
| CV-A6 | 26 (31%) | EV-C99 7/26 (26.9%)  E-11 3/26 (11.5%)  E-14 2/26 (7.7%)  E-7 2/26 (7.7%)  CV-A24 2/26 (7.7%) |
| EV-C99 | 21 (25%) | CV-A6 7/21 (33.3%)  CV-A2 3/21 (14.3%)  CV-A4 2/21 (9.5%) |
| E-11 | 10 (11.9%) | CV-A6 3/10 (30%)  CV-A4 2/10 (20%) |
| CV-A24 | 9 (10.7%) | E-21 3/9 (33.3%)  CV-A6 2/9 (22.2%)  E-14 2/9 (22.2%) |
| CV-A4 | 8 (9.5%) | E-11 2/8 (25%)  EV-C99 2/8 (25%) |
| E-14 | 8 (9.5%) | CV-A6 2/8 (25%)  CV-A24 2/8 (25%) |
| E-21 | 7 (8.3%) | CV-A24 3/7 (42.9%) |
| E-7 | 5 (6%) | CV-A6 2/5 (40%) |

* Enterovirus serotypes present in at least 5% of all samples with more than one enterovirus serotype detected
